# Bring me my alcohol!—On the continuum of pleasure and pain

**DOI:** 10.1111/nup.12403

**Published:** 2022-07-31

**Authors:** Regina Christiansen, Anette S. Nielsen

**Affiliations:** ^1^ Institute of Cultural Science, Faculty of Humanities, Philosophy University of Southern Denmark Odense Denmark; ^2^ The Faculty of Health Sciences, The Unit of Clinical Alcohol Research University of Southern Denmark Odense Denmark

**Keywords:** alcohol, conflicts, older adults, values

## Abstract

Alcohol use has been recognized as a challenge in eldercare and social care, and some anticipate that problems related to alcohol use will increase in the future as the current adult generation has high alcohol consumption rates. Accordingly, it is suggested that care workers are at risk of becoming passive bystanders to the destructive lifestyles of vulnerable older adults and even facilitating these lifestyles. In the present paper, we suggest that alcohol exacerbates and underscores inherent difficulties in eldercare, such as finding an appropriate balance between the personal freedom of the older adult and the responsibility of the care worker to provide care. The specific focus in the paper regard the communication and interaction involving values between people in eldercare in cases of problematic alcohol‐related situations to uncover the difficulties. We found it noteworthy that the objectives and perspectives of older adults, care workers, managers and relatives have implications regarding their interactions and communications because their varying experiences involve values that are not necessarily aligned. Sometimes, care workers have no choice but to act against what, in the public sphere and to the other care workers, is ruled out by virtue of their professional ethics. It is suggested that care workers describe and judge situations where alcohol is present paradoxically by virtue of their professional ethics, yet regulate their care to preserve the dignity of older adults, even when they find the situation to be an apparent dilemma.

## 1 INTRODUCTION

From health and economic perspectives, alcohol represents a major challenge on several levels. Around 100 million people worldwide are considered to have an alcohol use disorder (G. B. D. Alcohol Drug Use Collaborators, 2018), and alcohol is considered the most harmful drug globally (Nutt et al., 2010). Excessive alcohol consumption is the second most damaging lifestyle factor (after tobacco use), affecting the overall disease burden in high‐income countries (WHO, 2009). Thus, alcohol is a significant cause of noncommunicable diseases (Lopez et al., 2014). Given this fact, alcohol also represents a significant financial burden for society. The direct costs associated with alcohol account for 1.3% of the European gross domestic product (GDP; the indirect costs are twice that; Anderson & Baumberg, 2006).

However, simply listing the negative consequences of alcohol intake does not capture all aspects inherent in alcohol consumption. Individuals drink alcohol for various reasons; those reasons are rarely grounded in challenges and problems but have positive connotations. In Western societies, individuals drink alcohol to celebrate, socialise, relax, with meals or simply because it tastes good (Cunningham, 2012; Elmeland, 2015; Nielsen, 2003). For the general population, alcohol intake is not associated with illness, addiction and social burdens but rather with pleasure and quality of life.

## 2 ELDERLY AND ALCOHOL

At present, older‐age‐group individuals are retiring; this cohort has experienced economic growth in society and, for many, in their private lives compared to previous generations. Accordingly, they are the cohorts who grew up during a time when alcohol consumption per capita in many Western societies was increasing (Kuerbis, 2019; World Health Organization, 2014), for example, in Denmark (Elmeland, 2016). Current seniors and older adults regularly consume alcohol and are at a greater risk of developing alcohol problems than previous cohorts (Quinn & Mowbray, 2020). But what happens when individuals, who enter care homes or require home care, are from cultures where alcohol intake is associated with quality of life, well‐being, and pleasure and less connoted with a heightened risk of developing problems causing shame and stigma (Elmeland, 2015; McNeely et al., 2018)? Alcohol use has been recognised as a challenge in eldercare and social care, and some anticipate that problems related to alcohol use will increase in the future as the current adult generation has high alcohol consumption rates (Blow & Barry, 2012). In contrast to smoking, it is difficult to implement official policies for alcohol use. One reason may be that drinking alcohol is perceived as a private action that occurs in older adults’ homes (Harnett & Jönson, 2020, p. 6), even if they live in care homes. Therefore, the responsibility falls on care workers to handle difficulties or potential conflicts connected with the harmful effects of alcohol consumption. Research indicates that care workers consider alcohol consumption a ‘taboo’, and the management of related problems is complex (Oxholm et al., 2020). Additionally, relatives may facilitate the alcohol habits of older adults by delivering them alcohol even when drinking alcohol may conflict with medicinal indications. When care workers find themselves in situations where the alcohol consumption cause difficulties or additional workload, the care workers divulge that this leads to diffuse, uncomfortable experiences as they are expected to balance the care situation in compliance with their professionalism (Harnett & Jönson, 2020, p. 5). Sometimes, care workers are at risk of becoming passive bystanders to the life of the vulnerable older adults and even without intent facilitating unhealthy lifestyles (Eliasson‐Lappalainen, 1995). This points to a general premise in eldercare that care workers must constantly adjust their care practices to accommodate the diversity of older adults (Enßle & Helbrecht, 2020, p. 13).

## 3 RESEARCH DESIGN AND METHOD

The present paper discusses the difficulties associated with alcohol use by presenting excerpts of quotes based on qualitative interviews integral to the collaborative project Elderly Wellbeing and Alcohol‐a tricky cocktail (Klausen et al. 2020), which investigates quality of life among older adults in a Danish municipality. The prime research question addressed by the collaborative project is:

### 3.1 How can dealing with alcohol use in eldercare be improved to increase the quality of life of older adults and facilitate care workers?

The participants of the study pertained to older adults living alone with support from home care services, cleaning staff, home care residents, care workers, and relatives. At the institutional level, the research group included managers at the care homes. The care facilities were included based on their expression of interest and served a representative segment (i.e., socioeconomic background) of older adults in need of care and home care services (Klausen et al., 2020).

The facilities included (1) *Bakkegården* (Vejle centre), comprising 61 small apartments divided into 5 units for 61 older adults. *Bakkegården* is a typical full‐time care facility that accommodates older adults who are no longer able to manage their daily living activities for various reasons, including dementia and physical disabilities; (2) *Bakkeager* (7 km from Vejle centre), a care home consisting of five separate houses, each comprising 10 small apartments; most of its care workers have worked there for a long time; (3) *Gulkrog*, temporary accommodation for people requiring temporary intensive care (3–6 months) due to hospitalisation, the absence of relatives, the need to recuperate after surgery, or cannot take care of themselves or remain in their homes; (4) *Sofiegården*, an outbound home care service that includes nurses and care workers who provide personal, home care for older adults; and (5) *Hovergården*, a care facility that provides practical assistance for essential routine activities (e.g., dusting, cleaning the toilet and changing the sheets) in private homes (Klausen et al., 2020).

The project consists of four phases: exploration, interpretation (in collaboration with practitioners), development of a practice‐oriented model and an implementation process. Phase one was conducted in 2018; in this phase, observations were performed in the five care institutions. These observations led to the development of interview guides for older adults, care workers, relatives and managers, respectively. In total, 31 participants were interviewed for 30–60 min individually. Observations and interviews were written and transcribed, respectively, then analysed and interpreted by each researcher as part of their respective substudy in collaboration with five representative institutions (Klausen et al., 2020).

All participants gave their written consent to participate after they received both oral and written information about the study. Names given in this paper are all aliases to ensure anonymity. The study was processed on 22 February 2018 by the Danish National Research Ethics Committee.

## 4 CONTEXT AND AIM OF THE PAPER

Elderly care is considered one of the most fundamental care fields because of the vulnerability of older adults. Securing well‐being and quality of life may, at that stage, be reduced to ensuring basic needs rather than physical activities, outreach engagements and other aspects of life that require mobility, concentration and agility. In this context, alcohol, may play a role in promoting wellbeing of older adults because it is associated with pleasant meals and social interactions, albeit may also include attitudes, with multiple atypical value experiences of denial, fear, anxiety, taboo, uncertainty, insecurity, knowledge of prohibitions, ignorance, and so on. Alcohol in this context may therefore both be evaluated as a source of pleasurable moments but with the risk to transform into shame, guilt, distress and pain in older adults with consequences to the care workers ability to provide care. Such divergent attitudes have ramifications on how people in eldercare perceive and experience the use of alcohol in relation to well‐being, as well as the care workers ability to be decisive in various complex situations. In view of that alcohol exacerbates and underscores inherent difficulties, such as finding an appropriate balance between the older adults needs and wishes and the responsibility of the care worker to provide balanced care that is in accordance with her professionalism.

The present paper is a qualitative substudy of the collaborative project where the aim is to uncover the multiple value perceptions present in social interaction and communication among care workers, older adults, relatives and managers in eldercare in situations where the values comprise attitudes seemingly prototypical of cases where alcohol is predominant.

## 5 STUDYING VALUES IN ELDERCARE

To maintain and uphold caring practice, at times with a scarcity of resources, rapid change and new guidelines, it is necessary to clearly recognise what is involved in daily practice so that care workers can comply with values comprised of well‐being, health and working procedures (Banks, 2011, p. 13).

In general, values play a significant role in people's everyday dealings; in eldercare values such as dignity, compassion and respect, confer significance to the experiences of care workers and older adults. The philosopher and linguist Ray Jackendoff (2007) proposes that values are not ordinary objects, like physical objects, to which values relate in some way; instead, they are ‘a conceptualised abstract property connected to objects, persons and actions’ (p. 278). In accordance with such view the understanding is that values are involved when people in elder care ascribe something (an action, situation or object) with specific significance. An important element of the present approach is that the values are both ethical and relate to what is good or right (i.e., honesty, responsibility and respect); however, equally, values may relate to aesthetic experiences, epistemic states or pragmatic circumstances (for a similar description of the diversity of values see Morgan et al., 2015; pp. 25–26). For example, care workers may experience and describe a visit with an older adult as good, rewarding or difficult, depending on the care workers individual experience of a specific situation. Accordingly, for the present purpose the term ‘value’ denotes a property or aspect of a situation that is or is likely to be judged, perceived or felt to be (more or less) ‘good’ or ‘bad’, that is, an ‘evaluative aspect’ of the situation (Schroeder, 2016). The following quote expressed by a manager at a care home captures one such evaluation:

Personally, I believe…that great food tastes better with a glass of wine. Many people feel that way. There are also many here (older adults) who enjoy having a glass of wine (Interview: Karen, 2018).

Unfortunately, not all evaluations are as trouble‐free as expressed in the quote. In various situations, alcohol related issues may obscure the situation and care worker's ability to act upfront.

## 6 CONFLICT IN ALCOHOL‐RELATED VALUES

Often, values that relate to experiences where alcohol is present are not immediately available to the awareness of care workers. The values, nevertheless, have an acute impact on their perceptions and actions. Alcohol seems to be obscured by some inherited uncertainty that may easily prompt people to engage in and uphold pre‐established values. Some suggest an inherited taboo embedded in the concept of alcohol because of drinking‐related problems involving embarrassing, socially unacceptable, or life‐threatening behaviour and the consequences that follow (Elmeland, 2015).

A care worker was asked if she found alcohol‐consuming older adults challenging in her daily work. The care worker promptly replied that there is, in fact, an *alcoholic* in the care unit. Likewise, another care worker stated that ‘Birte is a former alcoholic, but she has not been drinking for the last thirty years’ (Interview: Anne, care worker, home care services, 2018). In these statements, the care workers evaluated an individual from the predicate of being an alcoholic or former alcoholic. The care workers evaluated and ascribed, possibly without intent, the older adult based on a personal normative value; viz., the older adult drinks alcohol problematically.

In these examples, the connotations of alcohol consumption are not associated with pleasurable moments to the drinking older adult but with pain, difficulties, suffering and, thus, do not initially relate to quality of life or well‐being in a positive sense, even if the consumption is terminated. Using such an approach, the well‐being of the older adult becomes a matter of controversy because of alcohol's influence on the normative concept of well‐being. Here, alcohol serves as an external component that comes between the older adult and the care worker. One consequence may be that the care workers come to ascribe the older adult in terms of values related to causing additional workload, detached from aspects of more prudential considerations of the older adult. When a care worker engages in this type of evaluation, she and her colleagues perceive the older adult as an alcoholic that requires targeted assistance to uphold a minimal sense of well‐being. Apparently, alcohol is circumscribed with some peculiarity that prompts care workers to evaluate the well‐being of older adults with problematic alcohol consumption less in terms of prudential characteristics. A care worker explains:

We have had a woman from Greenland…she is…in her early fifties where she is, we have known her for several years, and she is on her way to drink herself to death, she has an ostomy pouch (Interview: Tove, care worker, home care services, 2018).

In such situations, health‐related concerns are urgent to the extent that the older adult's drinking is negatively evaluated (viz. drinking herself to death). Furthermore, when an older adult practises excessive alcohol consumption that leads to oppressive demands of the care workers, alcohol becomes a problem and a hindrance to overcome; care workers must balance the wishes of the older adult with the preservation of their integrity by conforming to their professionalism.

A manager is aware of the potential distress alcohol‐related experiences have for the care workers and their ability to fulfil their professional duties. The manager explains:

That is when alcohol has, it is, of course, ehmm, some of the workers may feel very uncomfortable about entering those homes. Sometimes there might be one or more drinking buddies who are drunk, and there are vodka bottles and bottles of beer all over the place. It is difficult for them to know…that I have to perform my work procedures here. (Interview: Maria, home care services, 2018)

In such situations, there is a conflict between alcohol, as a personal matter of the older adult, and the alcohol consumption, which complicates the care worker's responsibilities. For older adults, pleasure is not simply a matter of their inner sense of well‐being but becomes an instance of well‐being as perceived and, more importantly, upheld by the care workers. In a different aspect we found an older male who had an alcohol consumption of 20–25 beers per day—he explains:

I like alcohol, I really do, right? I don't feel like involving others in my drinking. So…I don't drink while I am at work, right. It is…yeah, well, I don't know. I am not dependent. Ehhh…on alcohol, right (Interview: Hans, older adult, home care services, 2018).

Even though one might find 20–25 beers per day problematic due to the potential consequences of health, the older adult presents himself as a responsible person. He is aware not to involve others in his drinking by maintaining the consumption on a personal level. Subsequently, he mentions that alcohol serves as an entity of pleasurable experience:

Well…yeah…so we meet some old friends…they would like a beer. And then easily…then we easily connect. And then that day passed (Interview: Hans, older adult, serviced by home care, 2018).

Care workers express that delivery of care can essentially be described as getting the right help at the right time. Dilemmas occur when alcohol consumption increases the care worker's workload due to unhealthy use. In the aforementioned case, the older adult did not try to hide his alcohol consumption. Even though alcohol is objectively perceived and connected to unhealthy values, the older adult drinks alcohol as an experienced drinker because it offers him some positive affective experiences. When a care worker is asked if she thinks that alcohol might serve as a positive experience for the older adult, she mentions how aspects of vulnerability add to the complexity of the situation:

There are many vulnerable people, which influences our awareness, and then there is the alcohol, it is challenging to manage. If you feel annoyed…and one glass feels good…yeah, well, then maybe to consume is even better (Interview: Lotte, care worker, home care services, 2018).

She clearly considers alcohol to be a means of pleasure and easily becomes part of a problem when it is used to cope with mental challenges.

An older female living in her own home was capable to capture the need to balance one's alcohol consumption. She explains the rationale behind her and her family's alcohol consumption:

If we went out to dine or something like that, and accordingly, if we had parties at home, then we had wine for dinner, but it was not like we had to have it every day; it was only when we had guests or such (Interview: Bente, older adult, care home, 2018).

She places emphasis on balancing one's alcohol intake and not giving in to temporary temptations. She continues:

if one had to have…have alcohol and such every day, then I don't believe one would feel well‐being….I don't think so (Interview: Bente, older adult, care home, 2018).

From the female's perspective, alcohol would no longer be an entity associated with pleasurable moments. From her perspective, this would be an indicator of being unable to apply to one's own well‐being and changes into appreciating values that have no prudential significance. According to the female, the circumstances surrounding alcohol use and well‐being consist of a coherent combination of values, a combination of which she might not be explicitly aware of or able to justify. Nevertheless, she identifies her sense of well‐being as a long‐term ability to balance the consumption, considering the potential painful role of alcohol. She states that something initially appreciated for its prudential value may tip and become associated with values of negative characteristics.

## 7 ALCOHOL AS A BARRIER TO CARE

With the preceding consideration in mind, we anticipate that one of the main concerns of care workers is finding the right balance between the older adult's right to consume alcohol and securing his/her well‐being (e.g. preventing the older adult from having an accident, ensuring the older adult does not drink while on certain medication, or determining that the older adult is not drinking due to loneliness).

A manager explained how the care workers struggled to assist an older female adult limiting her alcohol intake; the woman suffered from severe alcohol dementia. Dementia and alcohol are considered unhealthy combinations (Gupta & Warner, 2008; Hislop et al., 1995), severely influencing older adult's ability to maintain well‐being. The relatives, however, are not in agreement with care workers regarding their recommendations; they (the relatives) complicate matters. The manager explains:

and…actually, we would have liked her to stop drinking, enabling us to assist her in a minimal sense, if one would like to get a minimal sense of her brain functioning to work…But the relatives showed pity towards her. It is about serving the beer every day; it was simply a nightmare. (Interview: Ditte, manager, temporary accommodation, 2018)

The care workers found themselves conflicted between values comprised to their professional expertise, the values of the relatives who felt bothered by the care workers suggestions, and the older adults whose drinking affected their dementia negatively. Obviously, the situation is complicated because care workers must employ their best knowledge of expertise and accordingly expected to act in favour of the older adult's well‐being and in the interest of the relatives. The older adult might have enjoyed drinking alcohol because it was appreciated as a resource value that offered the individual affective experiences irrespective of health concerns. The relatives may also consider alcohol a valuable resource value for its own sake and it tends to muddle their approach to care.

Another care worker explained how (s)he used his/her professional expertise to help an older male quit his alcohol consumption because his behaviour started to affect the well‐being of other older adults at the care home. This added yet another complexity to the situation. The care worker utters:

Well, several of us were involved, including the doctor and others, and he had talked to him about disulfiram^1^, and he (the older adult) had started to take disulfiram, but it was not his wish, so he drank while being on disulfiram… (Interview: Signe, care worker, home care services, 2018)

In the present context, disulfiram is presumably perceived as valuable because, in the long term, it would support the older male and the well‐being of the other older adults at the care home. Disulfiram and alcohol are diametrical entities, but both are considered a sort of value for the individual. For a drinking person, alcohol is valuable for its own sake; contrastingly, disulfiram is perceived as an unpleasant barrier. Disulfiram is beneficial after long‐term commitment and effort that may eventually offer the drinking person pleasurable experiences, albeit of a different kind (Swift et al., 1998).

The care worker was asked whether there might be other ways to solve the alcohol dilemma that caused problems for the drinking older adult, particularly regarding his surroundings and disulfiram, which is not only experienced as problematic by the older adult but does not even stop his drinking. The care worker could not imagine a solution. The care worker revealed how the older adult's autonomy influenced his chances to reduce his intake; the older male was fully obtaining alcohol by himself. The care worker considered if depriving the older adult alcohol would be of benefit to his well‐being. The care worker makes the following reflection:

well….at least not his well‐being. If I consider him. Because it is his wish, and his desire is to drink (Interview: Signe, care worker, home care services, 2018).

The care worker concluded that it would not benefit him if they prevented him from drinking. The care worker did not consider banning alcohol a solution because from the older male's perspective, well‐being equalled drinking alcohol. However, his drinking still decreased the well‐being of his surroundings. Thus, we recognise a conflict inhibited in such an endeavour because there may be a conflict between the affective value appreciation of alcohol to the older male and the value the care worker ascribes to alcohol as a merely perceived utility value related to the situation. The care worker approached the older male with support, she says:

So, it is all about….not supporting his drinking but supporting him in…maintaining those daily activities (Interview: Signe, care worker, home care services, 2018).

In this respect, the care worker does not deprive the male of drinking but, accordingly, for his sake, equally does not support him. Instead, she stimulates him with daily activities not involving alcohol.

Undoubtedly, care workers must navigate in far‐reaching dilemmas to which there are no straightforward solutions. We find that care workers at times tend to escape the difficult situations by uttering some general statements, for example, ‘but we must assist her at the level where she is at’ (Interview: Mette, care worker, home care services, 2018)…even if it, apparently, is not this simple.

## 8 INTEGRATING CONFLICTING VALUES TO PRACTICE

By now, it is evident that care workers often find themselves in difficult situations when alcohol is involved, situations that force care workers to balance divergent perceptions among older adults and their relatives while also mitigating their own perceptions. A care worker explains how two older adults got into a conflict over lunch because of diverging perceptions of drinking habits:

Once we had a citizen, who had a beer every day for lunch…and then another citizen said, “Oh you will end up an alcoholic if you need that beer every single day” (Interview: Jenni, care worker, care home, 2018).

For one older female, alcohol was appreciated for its affective value because she typically drank a beer with lunch every day. In contrast, the other female had a history of alcohol misuse; thus, she perceived alcohol with normative condemnation. She had been drinking for more than 30 years, albeit stopping when she entered the care home. With this in mind, the latter female should potentially be cared for differently than the former female. Enjoying a beer with lunch is, in isolation, uncomplicated to admit compared to not being allowed a beer for lunch. Care workers must balance those divergent values while accordingly applying pragmatic values at the institutional level (e.g., considering other older adults at the care home, regulations on alcohol, working procedures, etc.). When alcohol is evaluated negatively as problematic, it tends to infer the care workers’ ability to provide unconditional care because the drinking person's values fall short of the core values that care workers are expected to act in accordance with.

A care worker explained how it was not a problem per se when an older adult got drunk but more so that the older adult risked having an accident. She places emphasis to the following:

well, she is grown, and it is her choice; of course, it is. We let her drink if it pleases her because it is her home and her life. She has been like this for so many years. She is 82 already, so why should we try to change her habits? (Interview: Lone, care worker, home care services, 2018)

Care in such a case consists of ensuring that the female does not risk incurring an accident. One may argue that permitting the older female to drink as she prefers does not promote her prudential values because of the potential risk of an accident; one may even judge such an action as unprofessional. Nevertheless, even if one advocates for the care worker to behave in a manner that promotes the older female's well‐being, it is suggested that the care worker's actions display respect towards the older female. The care worker was not insensitive—she deliberates:

It is precisely due to a profound wish to assist people that you enter this profession. But I feel powerless. Every time she tries to quit drinking, her social life disappears, and she ends up alone. I find this to be a dilemma. Oppositely, however, I am aware of the harmful implication alcohol has to her. (Interview: Lone, care worker, home care services, 2018)

The care worker demonstrated a prerequisite of virtue in her job. She occasionally found herself in a dilemma where the most pertinent approach was to prevent herself from forming a one‐sidedly ethical judgmental attitude to cope with a situation and offer unconditional care.

Often, when care workers visit older adults, they find empty bottles, bags with empty vodka or rum bottles that have been left around for several days. The sight of empty bottles is ascribed as an aesthetic value that immediately prompts an affective value experience to the care worker. Even though they come to the older adult's home to fulfil some professional procedures, this pragmatic value can be overruled by other values. One care worker elaborates:

Well, one day when I had to do the cleaning, there were three to four people at the dining table, and they are having….actually we had changed the cleaning schedule so I could visit this citizen; they were sitting at the table, and he didn't know I was coming. Then I said that I would come back again the following day (Interview: Kirsten, care worker, home care services, 2018).

Care workers are fully aware that they enter the private home of the older adult, and for this reason, they must accept the older adult's living situation, even if the older adult begins drinking at nine o'clock in the morning. There is a distinction between when the older adults are around family or surrounded by friends. If friends are at the older adult's home and drinking alcohol, care workers must tell the older adults to ask their friends to leave. If the friends do not leave, the care worker is permitted to disregard the help. However, if family members are around, the care worker must continue with her tasks. The older adult may well consider his friends being over for a (alcoholic) drink as part of his well‐being; they drink together and socialise. Nevertheless, if the care worker interprets the situation as a hindrance to her ability to fulfil her professional tasks, she cannot assist the older adult to promote wellbeing. A manager conforms to a rather pragmatic approach to this challenging fact, she explains:

When someone requires assistance, you (older adult) don't have any privacy anymore. Then one has to acknowledge that we are professional workers, who do not interfere in their private lives, but we must help and support in all that is possible, and this is best done if…it is not preserving one's dignity if they have to cover up everything because we come calling all the time. (Interview: Karen, care home, 2018)

## 9 PHILOSOPHICAL RELEVANCE

During the study, it became apparent that alcohol is the source to multiple divergent value perceptions. The study reveals that care workers express multiple value perceptions that are prompted by difficult situations where alcohol is involved and where there is no immediate or straightforward solution.

In such deliberative acts where the care workers are required to give thought to appropriate care, the use of value perceptions involving thick and thin concepts play an important part of finding balanced solutions. Following Bernard Williams' differentiation between thin and thick as moral terms, one can distinguish between thin evaluative concepts, such as ‘good’ and ‘bad’, and thick (or substantive) evaluative concepts, such as ‘powerless’, ‘pain’ or ‘uncomfortable’. According to Williams, thick concepts are more specific than thin ones. Williams refers to the latter as ‘the most general expressions used in ethical discussion,’ and complains that these are the terms that theorists tend to favour (1985, p. 128). Judgments involving thick concepts are supposed to be both action‐guiding and world‐guided in the sense that the application of these concepts both depends on how things are and also determines what ought to be done. As Williams expresses it,'[t]he way these notions are applied is determined by what the world is like (for instance, by how someone has behaved), and yet, at the same time, their application usually involves a certain valuation of the situation, of persons or of actions. Moreover, thick concepts usually (although not necessarily directly) provide reason for action’ (1985, pp. 129–130). In contrast, judgments involving thin concepts are deemed merely action‐guiding—they tell us what to do but lack any descriptive content. It is noteworthy how care workers, when trying to do the right thing in situations, considered the many ways that non‐moral emotions shaped and interfered with their value judgements and experiences. In certain situations, alcohol was specifically perceived by care workers to exacerbate situations into value conflicts or what care workers themselves identified as so‐called dilemmas. Philosophically speaking dilemmas are situations in which there is a difficult choice to be made between two or more options, neither of which resolves the situation in a manner that is consistent with accepted ethical guidelines (see Sayre‐McCord [2013] for his perspective on dilemmas). In such difficult situations it is noteworthy how the care workers picked out evaluative concepts that are more or less specific. Initially, the care workers were likely to give sense of approval or disapproval of the specific situation, and thereby to communicate more significantly by the use of thin concepts. Notwithstanding, when the care workers were asked specifically about their point of view regarding the situation they communicated by the use of thick concepts and so more of a sense of what the situation were like to them so initially to be evaluated one‐sidedly by the use of thin concepts.

Such tensions occur when care workers form multiple value perceptions because of older adults’ alcohol consumption. Notably, care workers often experience multiple tangled emotions couched by thick value concepts related to ‘feelings of powerlessness’, ‘feelings of discomfort’ and ‘feelings of insecurity’ that are part of care workers awareness, conveying the implication that really solving difficulties takes time and effort. In situations where solutions made corresponds to the initial evaluation couched by thin concepts the solution, could potentially, lead to subsequent evaluations of normative significance, which, equally are couched in thin terms. In other words, the solution lacks information about what is truly occupying the minds of the care workers leaving them even more powerless.

Accordingly, it is apparent that when the care workers are required to act promptly and continuously balance numerous varying interests and concerns—principled ethics, be it in the form of traditional normative theories, such as deontology and utilitarianism, or domain‐specific approaches, such as bioethics (Beauchamp & Childress, 2001)—the theories seem to be of little help in easing the difficulties that care workers encounter every day. Principled ethics seemingly capture only parts of the dimension of the care workers' and older adults' moral lives that is of significance to them in situations when no immediate course of action is apparent. We suggest that alcohol is circumscribed with a peculiarity that prompt care workers to evaluate the situation at hand by thin value concepts. Consequently, in situations where solutions were offered these seemed to be based on thin concepts of ‘ought’ or ‘wrong’, with a risk of leaping to superficial solutions that were detached from the true significance of the problem at hand and for such reasons the situation may be experienced as a dilemma.

Tantamount to the philosophical account of dilemmas, it is evident that care workers have no choice but to act on what is otherwise excluded by virtue of their professional ethics. As demonstrated in the examples, the care workers had fairly ‘realistic’ expectations about solving dilemmas and accepted the ubiquity of dilemmas as an unavoidable part of care work. Paying overtly attention to ethical guidelines in difficult situations where feelings of discomfort and distress are upfront may constitute noticeable obstacles to moral knowledge or successful moral action as guidelines tend to rush care workers through their working routines, failing to pay attention to the modes of actions that would enhance the well‐being of older adults. When care workers get the time to reflect on their value perceptions, they immediately express how they feel and embrace suboptimal solutions with regard to some older adults (e.g., allowing alcohol consumption); these solutions, however, align with their professional integrity (viz: preserve dignity, respect individual differences, enhance wellbeing) and are in compliance with the individual older adult.

The present findings demonstrate that the authoritative role care workers assign to thin concepts is deeply entrenched in the way they (and the rest of us for that matter) engage, judge and express their experience of care situations and their ability to act appropriately. In situations where the reasoning and solutions provided involve thin concepts this exerts significant influence on the care workers ability to feel comfortable in the situation with the implication felt as being caught in a dilemma. By the same token, once the care workers acknowledge their feelings of distress, insecurity or powerlessness they are capable to use this information as an important element in reflections on the matter. In most situations, there are multiple thick value concepts available that can influence the deliberations and potentially might override the professional burden couched in thin concepts that the care worker is expected to conform to.

It is therefore suggested that care workers describe and judge situations where alcohol is present paradoxically by virtue of their professional ethics yet regulate their care to preserve the dignity of older adults, even when they find the situation to be an apparent dilemma. Being able to provide care when alcohol is present in a care situation reveals a care worker's ability to continuously re‐evaluate the values they might assign with prudential significance in favour of those significant to the older adult in front of them. Care workers often regulate their care and manage to solve such dilemmas by being open to the wishes of the older adult, meaning they do not approach the situation one‐sidedly with a definitive ethical attitude. When care workers succeed in remaining sensitive to the situation by refraining from applying an ethical attitude in a given moment it may to outsiders be perceived as a failure to comply with institutional guidelines. Nonetheless the remarkable fact is that when striving for an ethical and caring practice we may have to accept the peculiarity of moral practice. Even if, it is generally perceived to be a highly unsatisfactory and frustrating to be in these situations.

## 10 IN CLOSING

Through the involvement with the collaborative study, we became aware that the initial points raised by the district managers were significant. However, we also found that they did not completely capture the difficulties we encountered that were expressed by the individuals involved in the daily routines of eldercare.

A significant strength of present approach was that we investigated and analysed people's reality as it was understood through their value experiences and expressed in verbal and non‐verbal communications when they interacted and communicated in multiple situations. As part of the analysis, we also evaluated how experiences that involved values evoked emotions that impacted and amplified care worker's moral awareness of difficult situations (also see Faucher & Tappolet, 2002; Lance & Tanesini, 2004; Robinson, 2005). We found it noteworthy that the objectives and perspectives of older adults, care workers, managers and relatives have implications regarding their interactions and communications because their varying experiences involve values that are not necessarily aligned. Disarranged values may influence on the ability of care workers to follow their values, professional standards and ethics which is found to be strongly related to job satisfaction (Kramer & Hafner, 1989; Verplanken, 2004). Accordingly, such situations strongly influence the ability of care workers to handle feelings of distress (Slettebø & Bunch, 2004) and may lead to less integrity in their work (Glasberg et al., 2006; Sørlie et al., 2003), related stress and burnout (Juthberg et al., 2007; Wallin et al., 2015). Thus, there is a need to extend our understanding of the multiple value experiences and perceptions of people in eldercare as these can either motivate them in practice or cause negative feelings. As also uncovered in the present study, researchers have found that adhering too strictly to institutionalised guidelines and procedures can increase the risk of depersonalising the given care (López et al., 2021). Ethically difficult situations, from the perspective of care workers, is in general experienced to, and substantiated in the present study, to occur when they feel that they are unable to provide the quality care that corresponded with their ethical values (Glasberg et al., 2006; Rees et al., 2009). It is therefore suggested to be highly relevant and urgent to establish a formal space for discussions allowing care workers to re‐evaluate their actions and solutions as to comply with their professional integrity.

## CONFLICT OF INTEREST

The authors declare no conflict of interest.

## ENDNOTE

1 A drug used to support the treatment of Alcohol Use Disorder by producing sensitivity to ethanol
